# Herbaceous Dominant the Changes of Normalized Difference Vegetation Index in the Transition Zone Between Desert and Typical Steppe in Inner Mongolia, China

**DOI:** 10.3389/fpls.2021.832044

**Published:** 2022-02-07

**Authors:** Yanyan Lv, X. Q. Zhao, S. R. Zhang, J. G. Zhang, K. T. Yue, B. P. Meng, M. Li, W. X. Cui, Y. Sun, J. G. Zhang, L. Chang, J. R. Li, S. H. Yi, M. H. Shen

**Affiliations:** ^1^Institute of Fragile Eco-Environment, Nantong University, Nantong, China; ^2^School of Geographic Science, Nantong University, Nantong, China; ^3^Inshanbeilu Grassland Eco-Hydrology National Observation and Research Station, Beijing, China; ^4^Institute of Water Resources and Hydropower Research, Beijing, China; ^5^College of Urban Environment, Lanzhou City University, Lanzhou, China

**Keywords:** normalized difference vegetation index, unmanned aerial vehicle, species richness, shrub, herbaceous

## Abstract

Asymmetric responses of aboveground net primary productivity (ANPP) to precipitation were identified as a signal to predict ecosystem state shifts at temperate grassland zones in Inner Mongolia, China. However, mechanism studies were still lacking. This study hypothesized that the enhanced growth and newly emerged herbaceous after increased precipitation resulted in the highest asymmetry at the transition zone between desert and typical steppe. We monitored the responses of the normalized difference vegetation index (NDVI) of different species to precipitation events using un-manned aerial vehicle technology to test this hypothesis. NDVI and species richness were measured twice at fixed points in July and August with a time interval of 15 days. Results showed that: (1) From July to August, NDVI in the transition zone increased significantly after precipitation (*P* < 0.05), but NDVI in both the desert and typical steppe showed a non-significant change (*P* > 0.05). (2) In the transition zone, NDVI increases from the shrub and herbaceous contributed to 37 and 63% increases of the site NDVI, respectively. (3) There was a significant difference in species richness between July and August in the transition zone (*P* < 0.05), mainly caused by the herbaceous (Chenopodiaceae, Composite, Convolvulaceae, Gramineae, Leguminosae, and Liliaceae), which either emerged from soil or tillers growth from surviving plants. This study demonstrated that herbaceous dominant the changes of NDVI in the transition zone, which provides a scientific basis for the mechanism studies of ANPP asymmetric response to precipitation and warrants long-term measurements.

## Introduction

The grassland ecosystem is one of the main ecosystem types of terrestrial ecosystems, accounting for about 36% of the world’s land area and the residence of nearly 20% of the world’s population (Reynolds and Smith, 2002). Besides, it plays a crucial role in both carbon and water cycles. Under global change scenario, the precipitation pattern has significantly changed, and the grassland ecosystem sensitively responded to the changes of precipitation regime, especially for the arid and semi-arid regions (Knapp and Smith, 2001; Shen et al., 2018; Yang et al., 2020; Chen et al., 2021; Shen et al., 2021). In recent years, grasslands are experiencing frequent and intense droughts (Gidey et al., 2018; Cleland and Goodale, 2019), which inevitably cause changes in the function, structure and composition of the semi-arid and arid grasslands (Carlsson et al., 2017; Ochoa-Hueso et al., 2018; Stampfli et al., 2018). The catastrophic and irreversible changes may happen and cause a negative impact on human well-being (Riis et al., 2017).

Aboveground net primary production (ANPP) was influential in regulating ecological processes and the carbon cycle in arid and semi-arid grasslands. Its asymmetric response to precipitation has received lots of attention (Fang et al., 2001; Knapp and Smith, 2001; Knapp et al., 2017; Knapp et al., 2018). It was estimated as (ANPPmax – ANPPmean)/(ANPPmean – ANPPmin), a value of > 1 indicated that ANPP was reduced less in a dry year than it increased in a wet year, and vice versa (Knapp and Smith, 2001; Hu et al., 2018a). Hu et al. (2018a) indicated that the asymmetry increased first and then decreased with the increase of precipitation in the temperate grassland of China. The maximum asymmetry reached at the transition zone between desert and typical steppe, which has provided a critical warning signal to predict the stability and resilience of the temperate grassland ecosystems.

It was hypothesized that seedlings that emerged from the soil seed bank beneath bare ground strongly influenced the asymmetry (Holm et al., 2003; Li et al., 2008; Hu et al., 2018a). Lv et al. (2021) collected soil seed bank samples from the desert, typical steppe and the transition zone between both in Inner Mongolia, China, and incubated them in a laboratory. They found that both the soil seed bank density and similarity index between soil seed bank species and vegetation in transition zone were significantly higher than the desert and typical steppe. Moreover, herbaceous were the main components of the soil seed bank. The results proved that the herbaceous of the transition zone has potential influence on asymmetry. However, further quantitative studies on the response of ANPP to precipitation are demanded.

Several methods have been used to estimate grassland ANPP (Fang, 2004; Yu et al., 2017). The remote sensing inversion methods based on normalized difference vegetation index (NDVI) were relatively accurate in most environments (Seaquist et al., 2003; Emma et al., 2017). In some studies, NDVI was used to represent ANPP directly (Guo, 2013; Hu et al., 2018a). The NDVI values from satellite remote sensing usually have high temporal sampling but low spatial resolution, which prevented our further understanding of the contribution to NDVI changes from shrubs or herbaceous. Recently, the small un-manned aerial vehicle (UAV) technology has been extensively used in grassland species monitoring (Sun et al., 2018a; Wei et al., 2020), grassland aboveground biomass estimation (Zhang et al., 2018), with advantages of flexibility, low cost and a coverage range of satellite pixels. Meanwhile, the development of the FragMap system realized a long-term, repeated, and large-scale monitoring (Yi, 2017). Additionally, multispectral sensors can be easily applied to acquire high spatial resolution NDVI data (centimeter-level) (Deng et al., 2018; Mikhailova et al., 2020), which makes it possible to estimate the changes of ANPP from both shrub and herbaceous.

The objective of this study was to quantify the relative contribution of shrub and herbaceous to the NDVI changes in response to precipitation by using a UAV mounted with a multispectral camera. This investigation will provide evidence and scientific basics for revealing the mechanism of the asymmetric response to precipitation, and aid in diagnosing the responses of ecosystems to regional climate change and further understanding global climate change.

## Materials and Methods

### Study Area

**FIGURE 1:**
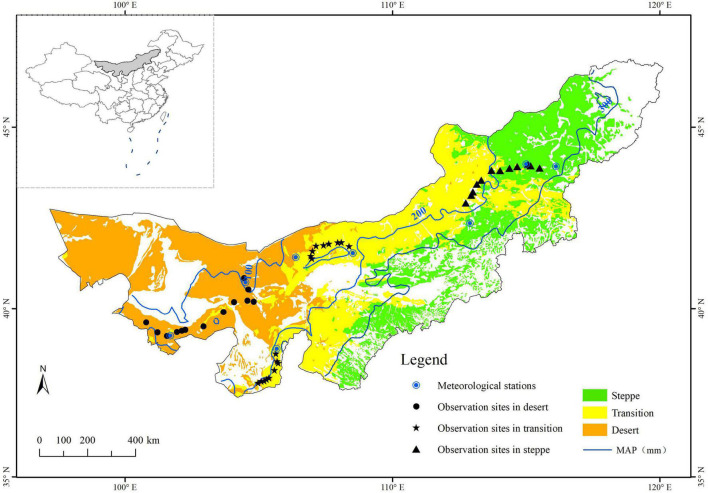
Location of the study area and sampling sites in the desert, transition zone, and typical steppe (Black dots, pentacles, and triangles denote the UAV observation sites in the desert, transition zone, and typical steppe, respectively. Bule circles denote the meteorological stations; MAP represents the mean annual precipitation).

This study was conducted in the desert, typical steppe, and the transition zone between both across Inner Mongolia, China (Figure 1). The study area is distributed along a mean annual precipitation gradient ranging from 100 to 300 mm, with 60–75% of the total annual precipitation in the peak growing season. The mean annual temperature ranges from –3 to 9°C. Soils shift from calcic brown/desert soils to chernozems and chestnuts. The vegetation types of this study are shrub (desert), shrub with herbaceous (transition), and herbaceous (typical). They belong to the grassland classes (1:1 Million Atlas of Grassland Resources in China, 1993) of the temperate desert, temperate steppe desert and temperate desert steppe, and typical steppe, respectively. Desert is characterized by short (10–25 cm) xerophytic species (mainly short shrubs and semi-shrubs) with sparse cover (15–45%) and low diversity (5–10 species in 1 m^2^). The steppe is more productive than the desert and has continuous vegetation cover. Relatively xerophytic tufted perennial grasses mainly dominate the vegetation. The plant communities have a height of 14–35 cm, 30–50% cover, and moderate levels of diversity (12–15 species in 1 m^2^) (Department of Animal Husbandry and Veterinary, Ministry of Agriculture [DAHV], and General Station of Animal Husbandry and Veterinary, Ministry of Agriculture [GSAHV], 1996; Hu et al., 2018a). The vegetation of the transition zone consists of both shrub and herbaceous (Wang et al., 2020). The diversity of species lies between the desert and typical steppe. According to Hu et al. (2018a), the asymmetry was highest in the transition zone while lower in the desert and typical steppe.

In our study area, precipitation was the main driving factor in the peak growing season (Guo, 2013; Hu et al., 2018a). Zhang et al. (2013) has also indicated a strong relationship between the precipitation and NDVI in the desert and typical steppe in during the peak growing season, while no relation between NDVI and temperature was found in Inner Mongolia. Therefore, this study mainly concerned the influence of precipitation on NDVI. The vegetation response to precipitation had an inevitable delay for 50–60 days (Cui et al., 2009; Liu et al., 2009). The daily precipitation data in 2020 obtained from the nearest meteorological stations and the mean cumulative precipitation from during observation was collected and shown in Table 1.

**TABLE 1:** The observation date and its cumulative precipitation for each type.

Types	Observation dates	Cumulative precipitation (mm)	Multi-year average precipitation (mm)
Desert	07.20, 08.06	2.82	115
Transition	07.24, 08.10	12.86	200
Typical	07.28, 08.15	69.61	300

The observation sites were strictly selected based on relative uniformity and representative of vegetation types. This study designed 13, 18, and 12 observation sites for NDVI and species monitoring in the desert, transition zone, and typical steppe, respectively (Figure 1).

### Field Observation

**FIGURE 2:**
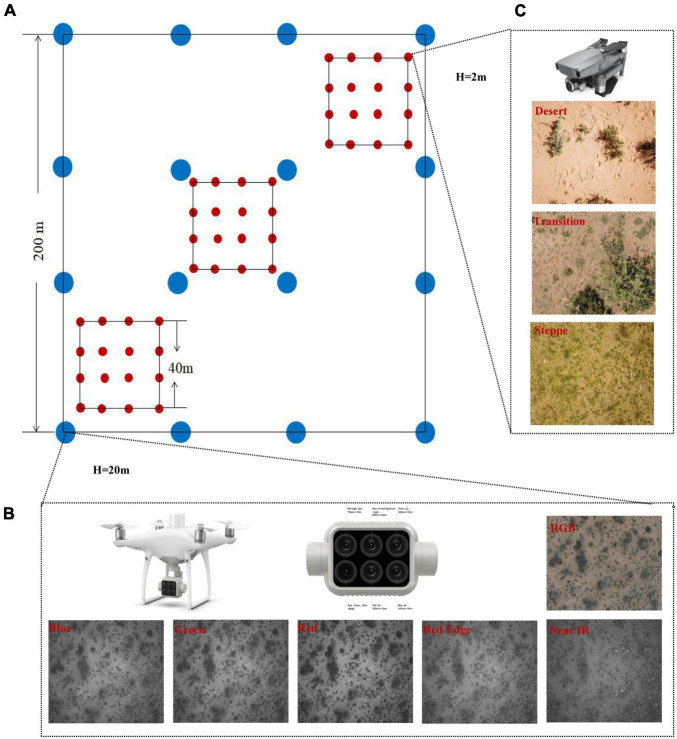
The strategy of data collection, UAVs, and aerial photos obtained. **(A)** strategy of the observation sites, there are 13, 18, and 12 observation sites for desert, transition zone, and typical steppe, respectively. In each observation sites, one GRID route and three BELT routes were designed. Blue dots are the way points of GIRD route (each way point has six multi-spectra aerial photos, defined as quadrat), while red dots are those of the BELT routes (each way point has one ordinary aerial photo); **(B)** is the Phantom 4 UAV, the multispectral camera, and aerial photos obtained in the transition zone, including RGB, Bule, Green, Red, Red Edge, and Near IR; **(C)** is the Mavic 2 zoom UAV, and aerial photos obtained from desert, transition zone, and typical steppe, respectively.

According to grassland growth status and spatial representatives, the range of 250 m × 250 m area was selected as a sampling site. Four flight paths were designed in each site, including one GRID route and three BELT routes (Figure 2A). The flight path of UAVs was set in FragMAP (Yi, 2017). Phantom 4 multispectral (manufactured by DJI Industries; http://www.dji.com) UAV was used to fly the GRID route (within the 250 m × 250 m area) at the height of 20 m with 16 way points (blue dots in Figures 2A,B). Three BELT routes were designed to collect species composition, and Mavic 2 zoom was used (within the 40 m × 40 m area) at the height of 2 m and 16 way points (red dots in Figures 2A,C). The positional accuracy of two UAVs is ±1.5 m horizontally and ±0.5 m vertically. Sixteen aerial photos were then automatically taken vertically downward in each route with one at each way point. The aerial photos resolutions of GRID and BELT were 1 and 0.09 cm, and their coverages were 26 m × 35 m and 3.43 m × 2.57 m, respectively.

The field observation was conducted in July and August. This observation time was consistent with the ANPP dataset used in Hu et al. (2018a). The first observation date for each type was shown in Table 1. For each sampling site, the repeated observation time interval was 15–16 day. In order to acquire the NDVI changes, several observations were conducted. We selected 4,128 multispectral aerial photos (for NDVI estimation) and 1,376 ordinary aerial photos (for species richness estimation).

### Reprocessing of Aerial Photos

Multispectral aerial photos were processed in the DJI Terra, ENVI (Version 5.3), and ArcMap software (Version 10.2) as the following steps: (1) the precise geometric correction and radiometric calibration were processed in DJI Terra, and the multispectral aerial photos were exported as tif format, with six bands included (RGB composite bands, Blue, Green, Red, Red Edge, and Near-Infrared) (Figure 2B); (2) NDVI values of multispectral aerial photos were calculated with the “Band Math module” in ENVI 5.3 and estimated as Eq. 1.

**FIGURE 3:**
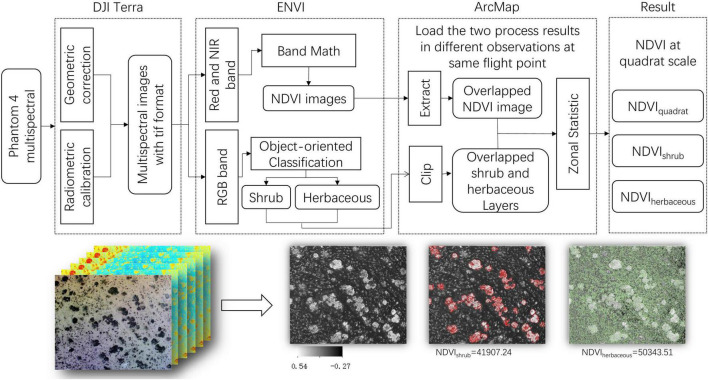
The schematic diagram of the reprocessing of aerial photos. NDVI_quadrat_ denotes the NDVI value of aerial photos, NDVI_shrub_ and NDVI_herbaceous_ denote the NDVI values of shrub and herbaceous, respectively.


Then the shrub and herbaceous were distinguished and classified based on human-computer interaction using the “Example-Based Feature Extraction Workflow module.” Each type of vector layer was exported as “shp” format. (3) The results (both the NDVI and vector layers in the same fixed flight point) were loaded into ArcMap together to extract the overlapped area. Finally, the statistical for NDVI value of the quadrat, shrub, and herbaceous were proceeded by the “Zonal Statistics module” (Figure 3).

(1) $$N\,D\,V\,I=\frac{N\,I\,R-R\,E\,D}{N\,I\,R+R\,E\,D}$$

where NIR and Red are pixel values in near-infrared and red channels, respectively.

The NDVI of observation sites (NDVI_site_) was calculated from NDVI_quadrat_ (Eq. 2). The percentage (%) of shrub / herbaceous NDVI value to observation site (P) was calculated with an average value of percentage in each quadrat (Eq. 3). The contribution (%) of the shrub / herbaceous NDVI changes to the site NDVI changes (C) was estimated as (Eq. 4).


(2) $$N\,D\,V\,I_{s\,i\,t\,e}=\sum_{i=1}^{n}\left(N\,D\,V\,I_{q\,u\,a\,d\,r\,a\,t.i}\right)/n$$

(3) $$\text{P}\left(\%\right)=\sum_{i=1}^{n}\left(\frac{N\,D\,V\,I_{h\,e\,r\,b\,a\,c\,e\,o\,u\,s.i}\,\left(N\,D\,V\,I_{s\,h\,r\,u\,b.i}\right)}{N\,D\,V\,I_{q\,u\,a\,d\,r\,a\,t.i}}\right)\times 100/n$$

(4) $$\text{C}\left(\%\right)=\sum_{i=1}^{n}\left(\frac{\Delta \,N\,D\,V\,I_{h\,e\,r\,b\,a\,c\,e\,o\,u\,s.i}\,\left(\Delta \,N\,D\,V\,I_{s\,h\,r\,u\,b.i}\right)}{\Delta \,N\,D\,V\,I_{q\,u\,a\,d\,r\,a\,t.i}}\right)\times 100/n$$

where *n* represent the number of quadrats (way points) in the same site, here *n* = 16, i represent the i-th quadrat, Δrepresent the subtract value between observation in July and August.

The NDVI, NDVI_shrub_, NDVI_herbaceous_, P, and C for desert, typical steppe and transition zone were represented by the average value of observation sites (13, 18, and 12 for desert, transition zone and steppe, respectively).

In this study, species compositions were identified visually based on aerial photos collected with Mavic 2 zoom at the height of 2 m. The species richness (species/m^2^) was then calculated as the species occurred in each aerial photo (Sun et al., 2018a).

### Data Analysis

An independent sample *t*-test was performed to analyze significant differences between the two observations. One-way ANOVA was used to test the significant differences among desert, transition zone, and typical steppe using SPSS 19.0 software.

## Results

### Normalized Difference Vegetation Index Value in Each Observation Site

**FIGURE 4:**
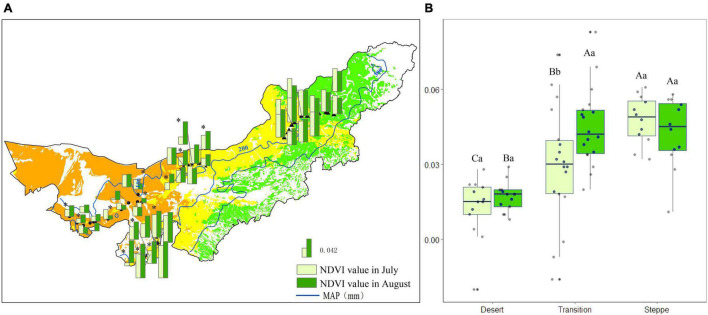
Normalized difference vegetation index value for each observation site **(A)**, and statistics for desert, transition zone, typical steppe **(B)** in July and August. Different capital letters denoted significant differences of NDVI value among desert, transition zone, and typical steppe (*P* < 0.05); while different lowercase letters denoted significant differences between July and August (*P* < 0.05); the same letters denoted non-significant differences (*P* > 0.05); MAP represents the mean annual precipitation. * denoted significant difference of normalized difference vegetation index value between the two observations of each site.

In desert, the mean NDVI value was 0.01 and 0.02 in July and August. Five out of 13 sites showed significant differences (*P* < 0.05) between the two observations (Figure 4A). The mean NDVI value in the transition zone was 0.03 and 0.04 in July and August. Nine out of 18 sites had significant differences (*P* < 0.05) (Figure 4A). The mean NDVI value was 0.04 for July and August in the typical steppe. The differences between July and August were not significant (*P* > 0.05; Figure 4A).

Normalized difference vegetation index value was the highest in the typical steppe, followed by the transition zone and desert; there was a significant difference among those three types in July (*P* < 0.05). A similar pattern was obtained in August. Nevertheless, there were no significant differences between typical steppe and transition zone (*P* > 0.05) (Figure 4B). Moreover, the NDVI value significantly differed between July and August in the transition zone (*P* < 0.05), while no significant difference was found in the desert and typical steppe (*P* > 0.05) (Figure 4B).

### The Percentage of Shrub and Herbaceous Normalized Difference Vegetation Index to the Site Normalized Difference Vegetation Index

**FIGURE 5:**
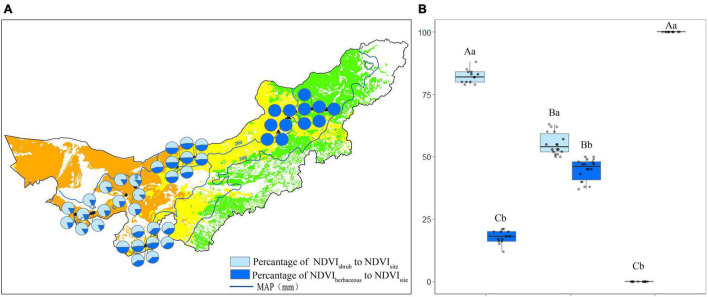
The percentage (%) of shrub/herbaceous NDVI to the site NDVI in each observation site **(A)**, and statistics for desert, transition zone, typical steppe **(B)** in July. Different capital letters denoted significant differences of the percentage of shrub/herbaceous NDVI to the site NDVI among desert, transition zone and typical steppe (*P* < 0.05); while different lowercase letters denoted significant differences between shrub and herbaceous (*P* < 0.05); the same letters denoted non-significant differences (*P* > 0.05); MAP represents the mean annual precipitation.

The percentage of shrub (P_shrub_) and herbaceous (P_herbaceous_) NDVI to the site NDVI in each observation site in July was shown in Figure 5. The P_shrub_ was 82%, while the P_herbaceous_ was 17% in the desert (Figure 5A). P_shrub_ and P_herbaceous_ were 56 and 43% in the transition zone, respectively (Figure 5A). In each site of the typical steppe, the P_shrub_ was 0%, while the mean P_herbaceous_ was 100% (Figure 5A).

The P_shrub_ was the highest in the desert (*P* < 0.05), followed by the transition zone and typical steppe. There was a significant difference among the three types (*P* < 0.05) (Figure 5B). The P_herbaceous_ was the highest in the typical steppe (*P* < 0.05), followed by the transition zone and desert; a significant difference among three types was also found (*P* < 0.05) (Figure 5B). For each type, the P_shrub_ significantly differed from the P_herbaceous_ (*P* < 0.05) (Figure 5B). Similar results were obtained in August.

### Contribution of Shrub/Herbaceous Normalized Difference Vegetation Index Changes to Site Normalized Difference Vegetation Index Changes

**FIGURE 6:**
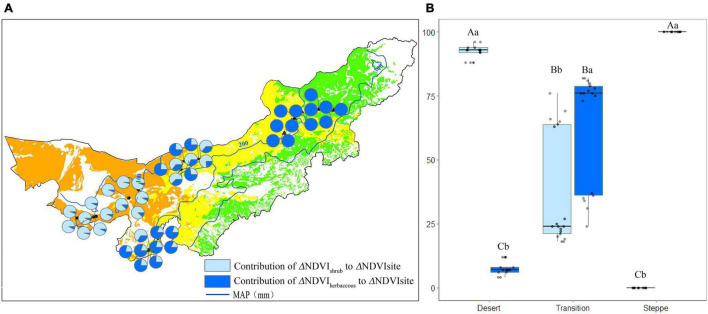
The contribution (%) of shrub/herbaceous NDVI changes to the site NDVI changes in each observation site **(A)**, and statistics for desert, transition zone, typical steppe **(B)**. Different capital letters denoted significant differences of the contribution of shrub/herbaceous NDVI changes to the site NDVI changes among desert, transition zone, and typical steppe (*P* < 0.05); while different lowercase letters denoted significant differences between shrub and herbaceous (*P* < 0.05); the same letters denoted non-significant differences (*P* > 0.05); MAP represents the mean annual precipitation.

The contribution of shrub (C_shrub_) and herbaceous (C_herbaceous_) NDVI changes to the site NDVI changes in each observation site was shown in Figure 6. The mean C_shrub_ was 92%, while the mean C_herbaceous_ was 8% in the desert (Figure 6A). The mean C_shrub_ was 37% in transition zone, while the mean C_herbaceous_ was 63% (Figure 6A). For each site of the typical steppe, the mean C_shrub_ was 0%; while 100% of the C_herbaceous_ (Figure 6A).

The C_shrub_ and C_herbaceous_ have differed in each type, i.e., C_shrub_ was significantly higher than the C_herbaceous_ in the desert (*P* < 0.05). At the same time, C_herbaceous_ was significantly higher than C_shrub_ in the typical steppe (*P* < 0.05) (Figure 6B). Although herbaceous NDVI account for a lower percentage of the site NDVI in the transition zone (Figure 5B), it is highlighted that C_herbaceous_ was significantly higher than that of the C_herbaceous_ in the transition zone (*P* < 0.05) (Figure 6B).

### Species Richness and Its Changes

**FIGURE 7:**
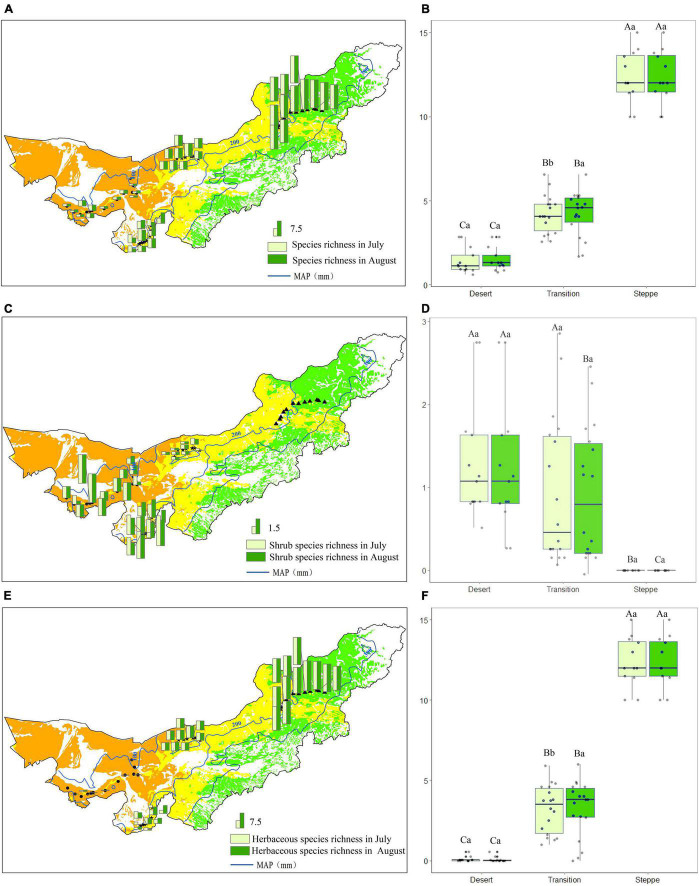
Species richness **(A,B)**, shrub species richness **(C,D)** and herbaceous species richness **(E,F)** in each observation site **(A,C,E)**, and statistics for desert, transition zone, typical steppe **(B,D,F)**. Different capital letters denoted significant differences of species richness among desert, transition zone, and typical steppe (*P* < 0.05); while different lowercase letters denoted significant differences between July and August (*P* < 0.05); the same letters denoted non-significant differences (*P* > 0.05); MAP represents the mean annual precipitation.

In the desert, the mean species richness was 1.4 and 1.5 species/m^2^ in July and August, respectively (Figure 7A). The mean species richness (species/m^2^) was 4.21 and 4.23 species/m^2^ in July and August in the transition zone (Figure 7A). For typical steppe, the mean species richness was 12.4 and 12.3 species/m^2^ in July and August, respectively (Figure 7A).

Species richness showed highest in the typical steppe (*P* < 0.05), followed by the transition zone and desert, there were significant differences among the three types (*P* < 0.05) (Figure 7B). For the same grassland type, species richness significantly differed in the transition zone between two observations (*P* < 0.05), while no significant difference in the desert and typical steppe (*P* > 0.05) (Figure 7B). Further analysis showed that shrub species have a non-significant difference in each observation (Figures 7C,D). Nevertheless, the herbaceous species richness showed a significant difference between the two observations in the transition zone (Figures 7E,F).

The aerial photos from 2 m hight showed that the herbaceous of *Agriophyllum squarrosum*, *Bassia dasyphylla*, *Convolvulus ammannii*, *Stipa capillata*, *Astragalus mongholicus* were not found in July, but seedlings emerged from the soil on the bare ground in August. *Artemisia frigida*, *Artemisia sieversiana*, *Cleistogenes squarrosa*, *Poa annua*, *Setaria viridis*, *Allium mongolicum, Stipa bungeana* were green in August, mainly from tillers growth for surviving plants (Table 2).

**TABLE 2:** Species and its changes between July and August in the transition zone.

Family	Species	Life-style	Types of species changes
Chenopodiaceae	*Agriophyllum squarrosum*	H; A	ES
	*Bassia dasyphylla*	H; A	ES
Composite	*Artemisia frigida*	H; P	ES/TG
	*Artemisia sieversiana*	H; A/P	ES/TG
Convolvulaceae	*Convolvulus ammannii*	H; P	ES
Gramineae	*Cleistogenes squarrosa*	H; P	ES/TG
	*Leymus chinensis*	H; P	ES
	*Poa annua*	H; P	ES/TG
	*Setaria viridis*	H; A	ES/TG
	*Stipa capillata*	H; P	ES
	*Stipa bungeana*	H; P	TG
Leguminosae	*Astragalus mongholicus*	H; P	ES
Liliaceae	*Allium mongolicum*	H; P	ES/TG

*H, herbaceous; A, annual; P, perennial; ES, Seedlings emerged from soil seed bank; TG, Tillers growth from surviving plants.*

## Discussion

### Significant Changes of Species Richness in the Transition Zone

Species richness was a simple and widely used index that indicated the diversity of a study area (Karen et al., 2004). In this study, there was a significant difference in species richness in the transition zone between July and August (*P* < 0.05), while non-significant differences were observed in the desert and typical steppe (*P* > 0.05) (Figures 7A,B). The particular precipitation characteristic, plant function traits, and water use efficiency may well explain these phenomena. The precipitation was one of the most critical factors limited plant growth in the transition zone, and was characterized by the small precipitation events (Guo, 2013; Zhang et al., 2013; Hu et al., 2018b). Not only shrubs but also herbaceous were observed in the transition zone. Non-significant differences in shrub species richness were obtained in our study (*P* > 0.05) (Figures 7C,D), which was consistent with Sun et al. (2018b). Cause most shrubs were phreatophyte, there was a weak response to the slight precipitation (Smith et al., 1997; Arndt, 2006). The shrubs were drought-tolerant, and they could obtain water (Soriano and Sala, 1983) and nutrients (Ogle and Reynolds, 2004) from deeper soil layers. There was a significant difference in herbaceous species richness (*P* < 0.05) (Figures 7E,F), which was consistent with Wu et al. (2019). Since the slight precipitation effectively increases the soil moisture precisely, and the herbaceous can use shallow soil water (Ehleringer et al., 1991; Dodd et al., 1998; Cheng et al., 2006). Further analysis showed both annual and perennial herbaceous changed sensitively with the precipitation (Table 2). The annual herbaceous included the *Agriophyllum squarrosum*, *Bassia dasyphylla*, *Artemisia sieversiana, Setaria viridis.* Those species mainly emerged from the soil seed bank due to plentiful seeds accumulated in the transition zone (Zeng et al., 2003; Lv et al., 2021). When the precipitation meets the germination requirement, plenty of seedlings emerges (Brown, 2003). They can quickly complete their life cycle (Liang et al., 2002; Li et al., 2006; Franks and Weis, 2008). The perennial plants included the *Artemisia frigida*, *Convolvulus ammannii*, *Cleistogenes squarrosa*, *Leymus chinensis*, *Poa annua*, *Stipa capillata*, *Stipa bungeana*, *Astragalus mongholicus*, *Allium mongolicum* (Table 2). On the one hand, the tillers from surviving plants used surface soil moisture effectively and grew quickly. On the other hand, the seeds stored in the soil seed bank also provided potential seedlings (Zeng et al., 2003; Lv et al., 2021).

### Contribution of Herbaceous to Normalized Difference Vegetation Index Changes Outweighed Shrub

Precipitation was the main driving factor in the peak growing season (Guo, 2013; Zhang et al., 2013; Hu et al., 2018b). Our study showed there were significant differences in NDVI value between July and August in the transition zone (*P* < 0.05), while no significant differences in the desert and typical steppe (*P* > 0.05) (Figure 4). The results indicated NDVI was more sensitive to the precipitation changes in the transition zone. The plant composition and environmental conditions were closely related to the results. In the desert, shrubs dominated, and the short-time precipitation could not be used effectively. Besides, the vegetation in this region was resistant to drought stress, reducing the NDVI sensitivity (Knapp and Smith, 2001; Bai et al., 2008). In the typical steppe, the vegetation was dominated by herbaceous, and the plant density was high. The competition among plant species becomes more critical, dampening NDVI sensitivity (Tilman, 1996; Bai et al., 2004; van Staalduinen and Anten, 2005; Hector et al., 2010). In the transition zone, species significantly changed with the precipitation (section “Significant Changes of Species Richness in the Transition Zone”), making a sensitive response of NDVI to precipitation.

A previous study indicated that the annual plants with short life histories can use a short period of precipitation to complete their life cycle in arid and semi-arid areas, and suggested promoting an increase of NDVI (Zhang et al., 2004). However, the quantitative studies were lacking. In this study, we have quantified that herbaceous contributed to 63% of the NDVI changes, significantly higher than the shrubs (37%) (Figure 6). It has been hypothesized that except for the increased growth of shrubs, the increased growth and newly emerged herbaceous might increase the NDVI in the transition zone (Holm et al., 2003; Li et al., 2008; Hu et al., 2018a). Our study has demonstrated that the NDVI changes from herbaceous with precipitation have significantly contributed to the site NDVI changes, either by new seedlings emerged in the soil seed bank or the tillers growth from the surviving plants.

### Mechanism of ANPP Asymmetric Response to Precipitation Requires a Long Term Monitoring

ANPP asymmetric response to precipitation was a critical warning signal for the shifts of the function and state of the grassland ecosystem (Hu et al., 2018a). Understanding the dynamic changes of ANPP and species composition is essential to reveal its mechanism. Understanding the dynamic changes of ANPP and species composition is essential to reveal its mechanism. Hu et al. (2018a) hypothesized that not only the growth of the plants, but also the increased number of new seedlings emerged from the soil seed bank influenced the asymmetry. Lv et al. (2021) found that the soil seed bank density and similarity index between soil seed bank species and vegetation in the transition zone were significantly higher than the desert and typical steppe. Moreover, herbaceous were the main components of the soil seed bank (Lv et al., 2021). The plenty of seeds can be emerged from the soil seed bank when the precipitation meets their germination requirement. However, it is unknown whether the seedlings of herbaceous can contribute more to the increase of NDVI than shrub. Our studies indicated that herbaceous dominants the changes of NDVI in the transition zone, either by the seedlings emerged from the soil seed bank, or tillers growth from the surviving herbaceous plants, which provided a scientific basis for the mechanism of the ANPP asymmetric response to precipitation. Nevertheless, the asymmetric responses are calculated based on the anomaly over long-term mean values. Therefore, long-term and high-resolution monitoring is required.

Grassland ANPP measurement was essential for revealing the mechanism of ANPP asymmetric response to precipitation. Field sampling, eddy covariance measurement and remote sensing inversion were the commonly used methods. The field sampling was accurate and straightforward, but it was time-consuming, laborious and destructive, and merely applied for a small area (Fang, 2004). The eddy covariance technique was temporal continuity and high spatial representativeness (Baldocchi, 2003; Chen et al., 2013, 2015; Yu et al., 2017; Hu et al., 2018b). Nevertheless, the coverage of flux footprint and the number of flux towers were limited. The remote sensing methods were based on the strong spectral signatures of plant canopy foliage in the visible and near-infrared bands (Craine et al., 2012; Edirisinghe et al., 2012; Li et al., 2014). Usually, NDVI was used to estimate ANPP indirectly in various spatial and temporal scales (Hu et al., 2010, 2018a; Yang et al., 2010). However, due to the low spatial resolution, the satellite remote sensing was difficult to distinguish NDVI from shrub and herbaceous. The UAV methods used in this study, when appropriately calibrated, may overcome these limitations. On the one hand, the UAV being mounted with a multispectral camera provided high resolution images (Guo et al., 2019), distinguishing the NDVI from shrubs and herbaceous (Figure 3). On the other hand, the FragMap system realized a repeated and long term monitoring (Yi, 2017), i.e., For each set of way points of GRID route, multiple flights with fixed height (20 m) could be executed at different times to conduct repeated monitoring of NDVI. Combined with BELT route (2 m), species monitoring is possible (Sun et al., 2018a; Wei et al., 2020; Meng et al., 2021). In our future studies, we will monitor dynamic changes in species and their NDVI over long-term period and over large regions, including the desert steppe, typical steppe, and the transition zone, to reveal the mechanism of the asymmetric response of ANPP to precipitation using a UAV.

### Limitation and Uncertainty

Our studies highlighted that herbaceous dominant the changes of NDVI in the transition zone in Inner Mongolia, China. Nevertheless, there exist some limitations and uncertainties in this study. (1) The aerial photograph was processed one by one, and lots of time and labors are needed. The batch data processing should be developed in future study. (2) Due to the differences in the precipitation regimes in each observation sites, the frequency of the observation should be high. The NDVI changes can only be monitored when the precipitation occurs. Besides, it was difficult to keep time synchronization at various observation sites. (3) Limited by the meteorological stations, it was a challenge to obtain the accurate precipitation data in each observation site. The amount of precipitation that caused the NDVI changes is still unknown. (4) Although precipitation is the main controlling factor of the ANPP in the Inner Mongolia, other biological and environmental factors such as temperature should be considered together in future studies.

## Conclusion

This study investigated the changes of NDVI and species richness at the fixed point by UAV in the desert, typical steppe, and transition zone between both in Inner Mongolia, China. The results showed that NDVI has a significant difference between July and August in the transition zone. The herbaceous NDVI changes contributed significantly to the site NDVI changes (63%). It was the main reason that caused the sensitive changes of NDVI in the transition zone to precipitation, which has provided the scientific basis for the mechanism of the ANPP asymmetry to precipitation. Nevertheless, high precision monitoring with UAV over a long-term period and over large regions still is needed.

## Data Availability Statement

The original contributions presented in the study are included in the article/supplementary material, further inquiries can be directed to the corresponding author/s.

## Author Contributions

YL, BM, and SY designed the experiments and manuscript draft. XZ, SZ, JZ, KY, MS, and WC processed the field observation and data analysis. ML, YS, JZ, LC, and JL revised the manuscript several times. All authors contributed to the article and approved the submitted version.

## Conflict of Interest

The authors declare that the research was conducted in the absence of any commercial or financial relationships that could be construed as a potential conflict of interest.

## Publisher’s Note

All claims expressed in this article are solely those of the authors and do not necessarily represent those of their affiliated organizations, or those of the publisher, the editors and the reviewers. Any product that may be evaluated in this article, or claim that may be made by its manufacturer, is not guaranteed or endorsed by the publisher.
